# Supplementary material for: Ohm’s law lost and regained: observation and impact of transmission and velocity zeros
Source: Nat Commun. 2024 Dec 5;15:10616. doi: 10.1038/s41467-024-54012-8 (PMC11621460; doi:10.1038/s41467-024-54012-8)
Supplement: Supplementary file 1 — Supplementary Information [file 41467_2024_54012_MOESM1_ESM.pdf]

Supplementary Information for "Ohm's law lost and regained: observation and impact of transmission and velocity zeros"

Krishna Joshi[1,2], Israel Kurtz[1,2], Zhou Shi[1,2,3] and Azriel Z. Genack[1,2]

[1]*Department of Physics, Queens College of the City University of New York, Flushing, New York 11367, USA*
[2]*Physics Program, The Graduate Center of the City University of New York, New York New York, 10016, USA*
[3]*OFS Labs, 19 School House Road, Somerset, New Jersey 08873, USA*

INDEX

Supplementary Notes

1. **Transverse electric and magnetic modes of cylindrical waveguides**

2. **Relationship between eigenchannel transmission, velocity, and surface energy density**

3. **Undoing the impact of absorption upon measurements of absorbing samples**

4. **Determination of the experimental signal to noise ratio**

5. **Impact upon transmission time by transmission zeros in medium with gain or loss**

Supplementary Figures

Fig. S1. Measurements of transmission zeros for different lengths

Fig. S2. Impact on the conductance of the increasing spectral range of transmission zeros with sample length

Fig. S3. Undoing the impact of absorption in experimental samples

Fig. S4. Measurement of signal to noise ratio

Fig. S5. Lorentzian fit to the transmission zeros

Fig. S6. Fluctuations in conductance in measurements and simulations

Fig. S7. Computational geometry

Fig. S8. Noise in transmission eigenvalues in samples with different N

Fig. S9. Effect of spatial resolution on crossovers

Fig. S10. Measured conductance and density of states

Fig. S11. Correlation between transmission eigenvalues

Fig. S12. Impact of TZs on conductance in single configurations

References

Supplementary Notes

1. Transverse electric and magnetic modes of cylindrical waveguides

A cylindrical waveguide with radius a and perfect metallic boundary (PMB) conditions supports two modes at each group velocity. The electric field is transverse to the propagation direction for transverse electric modes, while the magnetic field is transverse to the propagation direction for transverse magnetic modes. Due to the rotational symmetry of the waveguide, the solution of Maxwell equations in cylindrical coordinates (ρ, φ) can be written as $\psi(\rho, \varphi) = \psi(\rho)e^{in\varphi}, n = 0, 1, 2, \ldots$. The solutions of the wave equation for the cylinder is the solution of Bessel's differential equation[1]

Transverse electric modes:

$$E_\rho = \frac{j\omega\mu n}{k_c^2\rho}[Acos(n\varphi) - Bsin(n\varphi)]\, J_n(k_c\rho), \qquad E_\varphi = \frac{j\omega\mu}{k_c}[Asin(n\varphi) + Bcos(n\varphi)]\, J_n'(k_c\rho)$$

Transverse magnetic modes:

$$E_\rho = \frac{-j\beta}{k_c}[Asin(n\varphi) + Bcos(n\varphi)]\; J_n'(k_c\rho), \qquad E_\varphi = \frac{-j\beta n}{k_c^2\rho}[Acos(n\varphi) - Bsin(n\varphi)]\, J_n(k_c\rho)$$

Here, J_n and J_n' are, respectively, the nth-order Bessel function of the first kind and its derivative. The integer n represents the periodicity of the solution in φ. The PMB boundary conditions at $\rho = a$ give $J_n'(k_c a) = 0$ (transverse electric), $J_n(k_c a) = 0$ (transverse magnetic). If the roots of J_n are defined so that $J_n(p_{nm}) = 0$, where p_{nm} is the root of J_n, then k_c must have the value $k_c = p_{nm}/a$. The propagation constant for a transverse magnetic mode with indices (n, m) is given by $\beta_{nm} = \sqrt{k^2 - k_c^2}$, where $k = 2\pi/\lambda$ is the wave number. The condition $\beta_{nm} > 0$ determines the maximum number of propagating modes in the waveguide at a given frequency.

The total number of propagating waveguide modes in our experiments is 62 before the crossover and 64 after the crossover at 14.8317 GHz. Fig. S1 shows spectra of transmission eigenvalues for n = 61, 62, 63, and 64 for lengths L = 23, 40, and 61 cm. The left and right panels show two different sample realizations obtained by rotating the copper tube about its axis. Fig. S2 shows the simulated results of the impact on conductance with sample lengths.

In the experiment, we found a considerable drop in conductance for the crossover N = 61, 62 to 63, 64. However, in our simulated model[2,3], the dip is almost non-existent for N ≥ 40. This behaviour can be attributed to the geometry of our experimental system (3D) and simulated model (2D). The cross-section of a 3D system is larger; hence, the number of propagating modes is greater than that of a 2D system at a given frequency.

2. Relationship between eigenchannel transmission, velocity, and surface energy density

The basis function corresponding to the mth mode of the empty waveguide is φ_m. Then the field coefficient of transmission for the field in position space is the Greens function G. G is projected

into the space of waveguide modes according to $t = 2i\varphi\ e\ \varphi^{\dagger}$, where t is now the transmission matrix in the basis of waveguide modes and φ is the vector of m waveguide modes.

The singular value decomposition of transmission matrix in the space of waveguide modes is: $t = U\Lambda V^{\dagger}$, where V and U are singular matrices corresponding to the input and output surfaces, respectively. The transmission eigenvalues τ_n are the squares of the singular values, which are the diagonal elements of Λ: $\tau_n = |\lambda_{nn}|^2$. The eigenchannel velocities v_n are determined by $\frac{1}{v_n} = \sum_m \frac{1}{\mathrm{v}_m} |u_{nm}|^2$, where u_{nm} is an element of U and v_m is the group velocity of the mth waveguide mode. The eigenchannel energy densities u_n are given by $u_n = \sum_m \frac{1}{\mathrm{v}_m} |t_{nm} v_{mn}|^2$, where t_{nm} is an element of t and v_{mn} is an element of V. Since $tV = U\Lambda$, this implies that $u_n = \sum_m \frac{1}{\mathrm{v}_m} |\lambda_{nm} u_{mn}|^2 = \tau_n \sum_m \frac{1}{\mathrm{v}_m} |u_{nm}|^2 = \frac{\tau_n}{v_n}$.

3. Undoing the impact of absorption upon measurements of absorbing samples

The impact of absorption on measurements of field transmission coefficients is compensated for by applying the following procedure with reference to Supplementary Fig. S3[–7]:

1. Each data point of the raw spectrum (a, blue curve) is multiplied by a Gaussian cantered at that point (Supplementary Fig. S3a, black dashed curve).
2. The spectrum is then Fourier transformed into the time domain (Supplementary Fig. S3b, blue curve)
3. The signal in the time domain is then multiplied by $e^{\gamma_{\mathrm{g}}}$ where γ_{g} is the added gain. The time-domain signal is then cut off at a time delay at which the noise is comparable to the signal (b, red curve) to reduce the impact of noise in the Fourier transformed spectrum.
4. The signal is then Fourier transformed back to the frequency domain to produce the modified spectrum in which added gain compensates absorption in the sample (Supplementary Fig. S3a, red curve).

The probability distributions of the intensity transmission coefficient obtained in this way are in accord with predictions for a sample without absorption[4]. We note that this process produces a spectrum that is the same as would be obtained from a superposition of modal partial fractions with the linewidth of all modes reduced by γ. However, because of the nonuniform distribution of gain within the medium, different quasi-normal modes have slightly different rates of decay due to absorption so that absorption cannot be perfectly cancelled at all frequencies by this method.

4. Determination of the experimental signal to noise ratio

Once the measurement of the TM is completed, a measurement of the field spectra (t_{ba}) at the first position and polarization is repeated so that the signal to noise ratio in the experiment can be determined. The field intensity spectra are measured from the square of field coefficient, i.e., $I = |t_{ba}|^2$. In Fig. S4, measurements of the first intensity spectra (signal) are compared to the spectra taken after the end of the measurements (reference signal). Top panel represents the difference between these spectra while the bottom panel is a comparison between them. The signal to noise ratio is calculated from the ratio $\sigma_I / \bar{I}$. σ_I is calculated from the spectrum of the difference of

each of the two spectra from their average $\bar{I}$, $\sigma_I = \sqrt{\left\langle (\frac{\Delta I}{2})^2 \right\rangle}$. The average value of signal to noise ratio is 280.

The principal factors that determine the SNR in our experiments are the variation with temperature of the gain of the amplifier and of the structure of the sample, and the accuracy of the positioning of the source and detection antennas. Since the transmitted field is a result of interference of partial waves following trajectories of different lengths, changes in path lengths of transmitted waves with temperature lead to changes in the transmitted intensity. The interference of waves is affected by increasing temperature via the expansion of the elements of the sample of alumina spheres in Styrofoam shells in air contained in a copper tube. This is partially compensated for by the associated reduction in the indices of refraction of media of lower density as temperature increases. In addition, the changes in the sample lead to changes in scattering, and so in the distribution of wave trajectories.

The typical pathlength of transmitted waves may be found from the average transmission time through the 23-cm-long sample given by the average of the phase derivative, $\langle t \rangle = \langle d\varphi/d\omega \rangle = 34$ ns. Since sharp resonances do not occur over the spectral range of the measurements, the average refractive index may be taken as the average of the indices of refraction weighted by their volume faction, to give an average index of $n = 1.15$. This gives an optical path length of $\langle c/n \rangle \langle t \rangle = 8.9$ m. These factors result in a change in the partial waves and the interference between them, which cannot be calculated readily, but likely contributes to the variation of spectra over time. Another possible source of noise in the measurement of the TM is shifting of the sample, but this seems to have been eliminated by shaking the sample before the measurements are made. Finally, the control of the position of the source and detector antennas are not perfect. Most of these factors can be improved with better control of the temperature and positioning. Nonetheless, it was possible to reliably obtain spectra of transmission eigenvalues because of the correlation between the transmission eigenvalues.

5. Impact upon transmission time by transmission zeros in medium with gain or loss

In the presence of loss or gain, TZs on the real axis in the complex plane are moved off the real axis. If the rate of the loss (gain) of the field of γ $(-\gamma)$ were the same for all quasi-normal modes, the poles would be displaced vertically in the complex plane by γ (-γ). Since the field is due to the sum over modal contributions, the TZs should be displaced to the same degree. This would give rise to Lorentzian lines in t_N with peaks (dips) of $1/\gamma$ $(-1/\gamma)$ and half widths $|\gamma|$, in accord with equation (2). Transmission time τ_{T} is the sum of the transmission time of all the TEs, $\tau_{\mathrm{T}} = \sum_n^N t_n{}^4$. τ_{T} is the sum of Lorentzian functions associated, respectively, with the poles, τ_{p}, and the zeros, τ_{z}. Hence, each TZ's dips in the t_n will have a Lorentzian line shape. Fig. S5 represents a Lorentzian fit to the 3rd TZ in the simulated spectrum of t_7 with the added absorption $\gamma_a = 3 \times 10^{-4}$. The Lorentzian fit function is defined as $L(\omega) = -\zeta_i/[(\omega - Z_i)^2 + \zeta_i{}^2]$. Z_i and ζ_i represent the position and half width of the Lorentzian, respectively.

It is evident from Fig. 5e, however, that the dips in t_N are not all equal. This indicates that the narrowing or broadening of quasi-normal modes, and the corresponding displacement of the poles up or down in the complex plane by gain or loss, differ for different modes. The

transmission time $t_{N=62}$ for the experimental configuration shown in Figs. 2a-d for the raw data and for three different values of added gain, γ_g, is shown in Fig. 5f. Gain is added to remove the impact of absorption. The field transmission spectrum is Fourier transformed into the time domain. The time signal is then multiplied by $e^{\gamma t}$[7]. The signal at late times is set to zero because the noise is amplified by the added gain (Fig. S3). Dips and peaks occur in t_{62} at the same frequencies at which dips occur in $\log \tau_N$. The variation of spectra of t_{62} with gain or loss shows that these dips are due to TZs near the real axis of the complex plane. The noise in the measurement is greater than in measurements of $\log \tau_N$ because of the greater accuracy needed to calculate the spectral derivative of the field involving difference in the phase of the wave at two frequencies. The accuracy of the determination of the phase derivative from measurements is also compromised by the size of the frequency steps of 300 kHz.

Spectra of the time delay for $N = 62$ are displayed in Fig. 5f for the raw data of the moderately absorbing sample and for added gain of γ_g = 0.002 (red), 0.005 (blue), and 0.02 ns^{-1}(pink). The four numbered features in these spectra correspond to the four numbered dips in $\log \tau_n$ in Fig. 2b. Features 1, 3, and 4 are negative for the raw data and positive at the highest level of gain. These correspond to single TZs moving from the lower to the upper half of the complex plane as the gain level increases, in accord with the expression for τ_z in equation (2). In the third numbered feature, the depth of the dip first increases as gain increases before this feature becomes a peak. This indicates that the TZ first moves closer to the real axis as gain is added, and then is pushed above the real axis at the highest level of gain. In the second feature, the raw data shows a peak in the time delay. This corresponds to the case of a conjugate pair of TZs being lowered by absorption, bringing the upper TZ closer to the real axis than the lower TZ[6]. This gives a peak in t_{62} for the raw data which is transformed into a dip as the lower TZ approaches the real axis.

Supplementary Figures

Fig. S1 | Measurements of transmission zeros for different lengths.

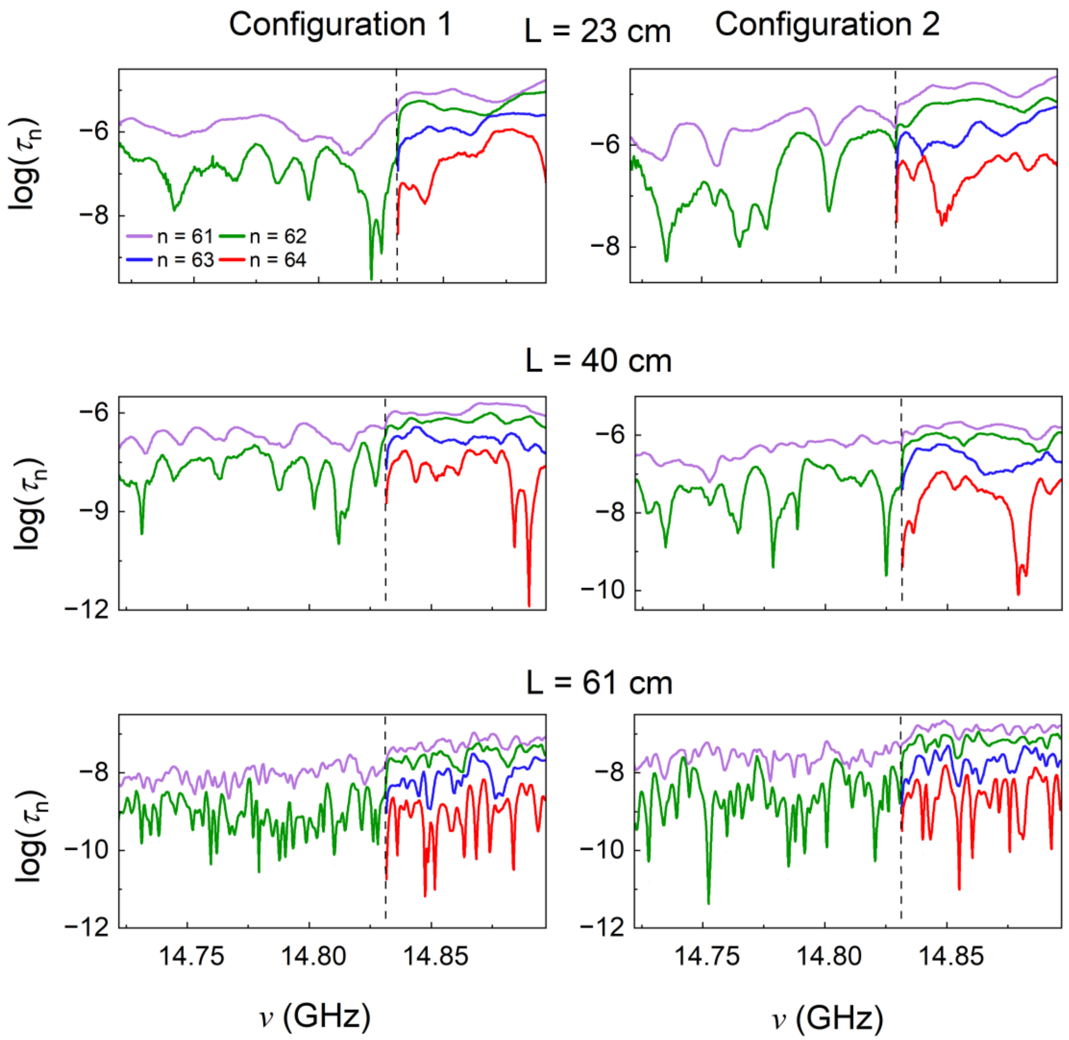

Spectra of the logarithm of transmission eigenvalues for n = 61, 62, 63, and 64 for sample lengths $L =$ 23, 40, and 61 cm. The left and right panels present results for two different disordered configurations. The lowest value of τ_N in the spectra shown is of order of nine. This is nine orders of magnitude below the noise level in the experiment. Aside from noise in the measurement, the lowest value of τ_N is limited by the closeness of the frequency at which the measurement is made to the frequency of the TZ, and by the nonuniformity of absorption in the medium once the average absorption level is compensated for by adding gain using the approach illustrated in Fig. S3.

Fig. S2 | Impact on the conductance of the increasing spectral range of transmission zeros with sample length.

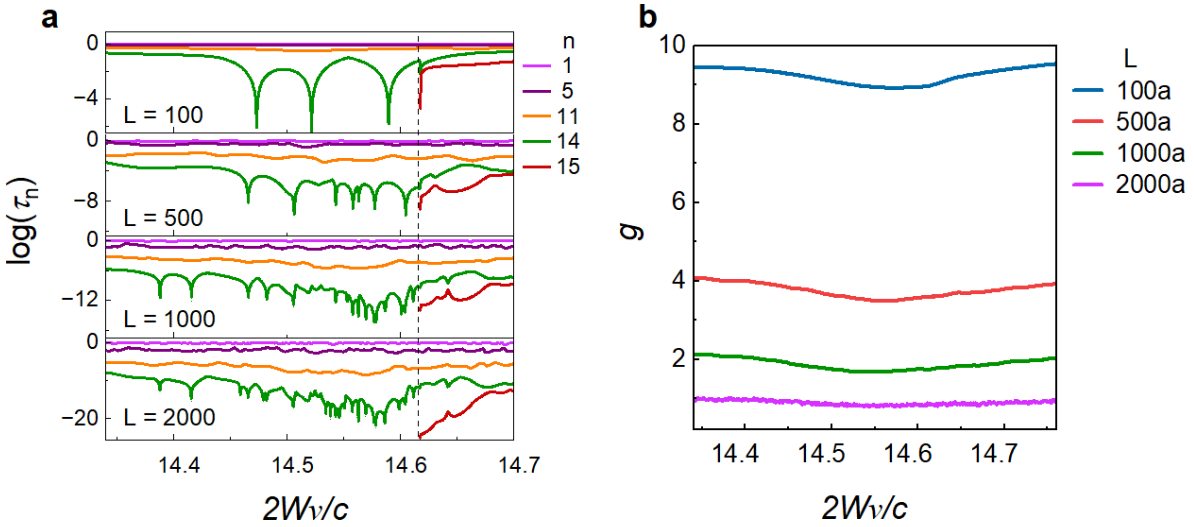

a, The spectral range of TZs near the crossover from $N = 14$ to $N = 15$ increases with sample length for individual configurations of width, $W = 60a$. **b,** The dip in the conductance washes out as the length increases as the spectral range of the TZs increases.

Fig. S3 | Undoing the effects of absorption in experimental samples.

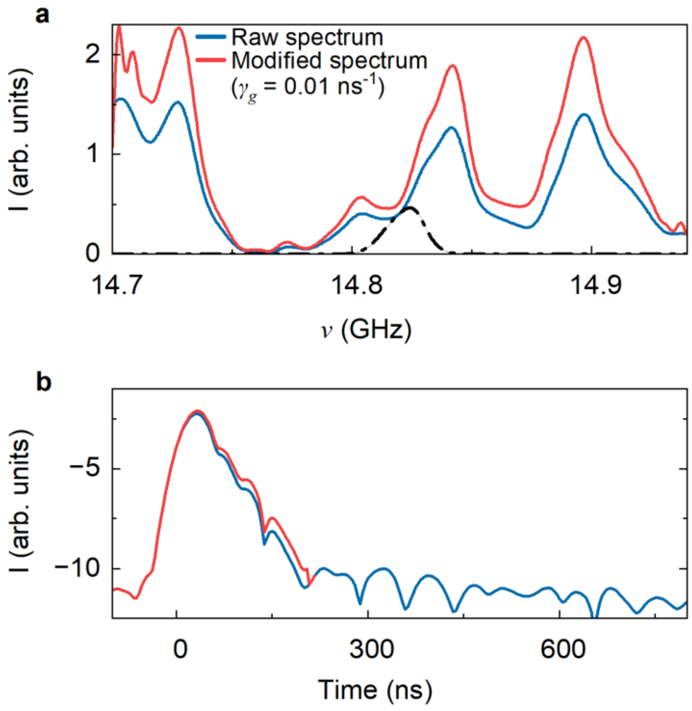

Undoing absorption in the experimental samples: **a,** Raw spectrum (blue curve) and modified spectrum after adding the gain $\gamma_g = 0.01\ ns^{-1}$ (red curve). The dotted black line represents the Gaussian pulse, whose Fourier transform corresponds to the incident pulse whose time response is computed. This makes it possible to act on a small portion of the raw spectrum. **b,** Fourier transform of the raw spectrum (blue curve) and modified spectrum (red curve).

Fig. S4 | Measurement of signal to noise ratio.

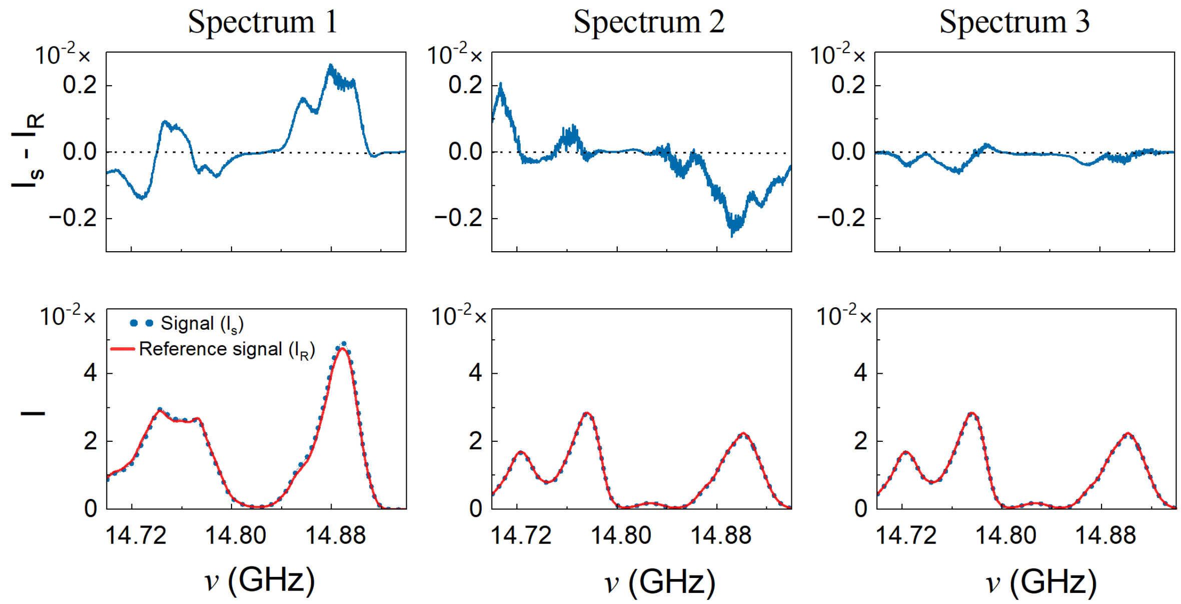

Top panel: Difference between the measured intensity spectrum (signal) and the spectrum at the end of the measurement (reference signal) for three different spectra. Bottom panel represents a comparison between the signal (scatter blue curve) and the reference signal (red curve). The field intensity spectra are measured from the square of field coefficient, i.e., $I = |t_{ba}|^2$. The signal to noise ratio is calculated from the ratio $\sigma_I / \bar{I}$. σ_I is calculated from the spectrum of the difference of each of the two spectra from their average $\bar{I}$, $\sigma_I = \sqrt{\left\langle (\frac{\Delta I}{2})^2 \right\rangle}$. The average value of signal to noise ratio is 280.

Fig. S5 | Lorentzian fit to the transmission zeros.

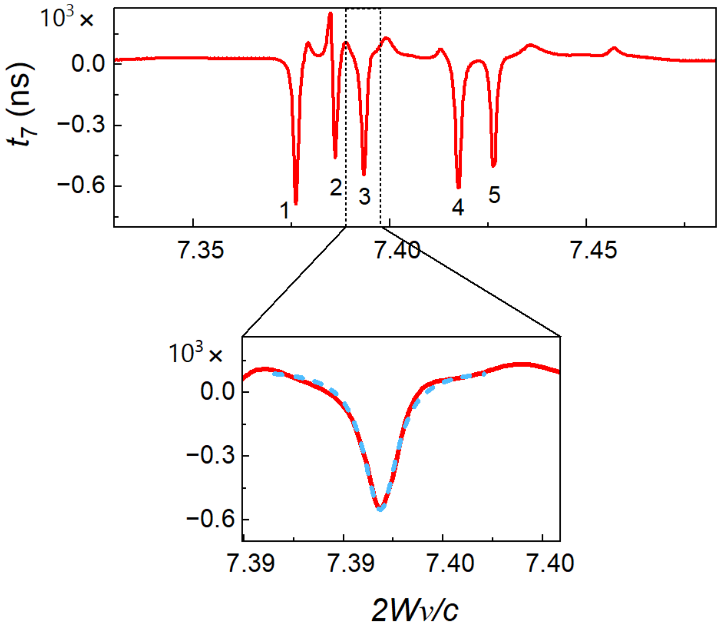

A Lorentzian fit to the 3rd TZ (dotted cyan curve) in the simulated spectrum of t_7 (solid red line) with absorption rate $\gamma_a = 3 \times 10^{-4}$.

Fig. S6 | Fluctuations in conductance in measurements and simulations.

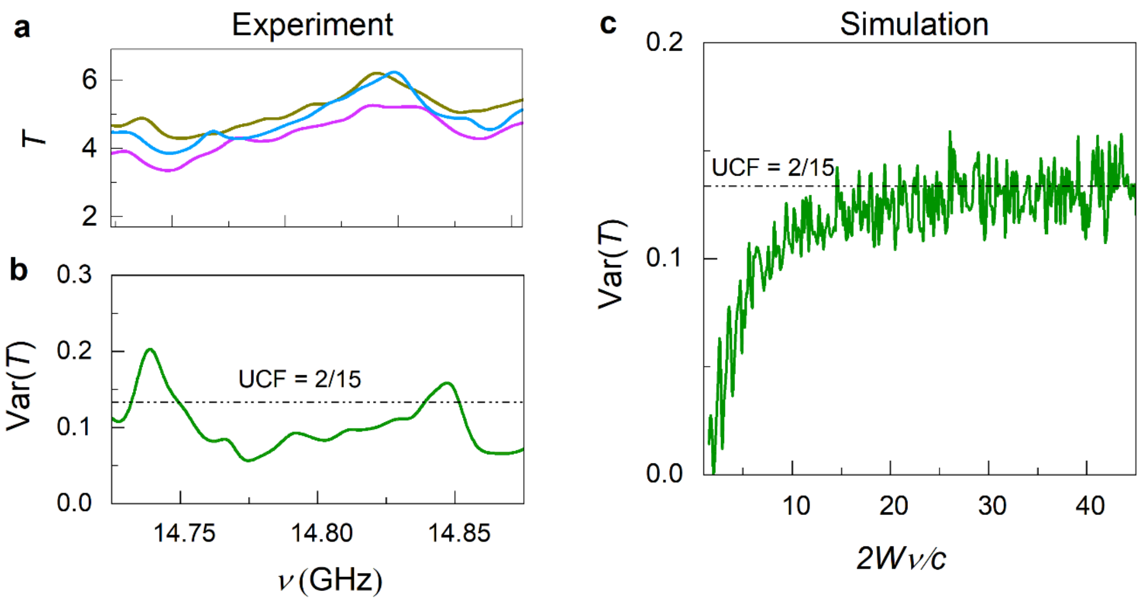

a, Measurements of the transmittance in three disordered configurations with $L = 23$ cm. **b,** Measurements of $\mathrm{var}(T)$ for the random ensemble of 23 configurations. The value of $\mathrm{var}(T)$ fluctuates around the value predicted for UCF of $2/15$. **c,** Results of simulations over 500 sample configurations of $\mathrm{var}(T)$ as a function of sample width at fixed length $L = 1000a$ for the same samples for which results are shown in Fig. 6d. As W increases, bringing the sample into the diffusive regime with $g > 1$, $\mathrm{var}(T)$ approaches the predicted value of UCF of $2/15$.

Fig. S7 | Computational geometry.

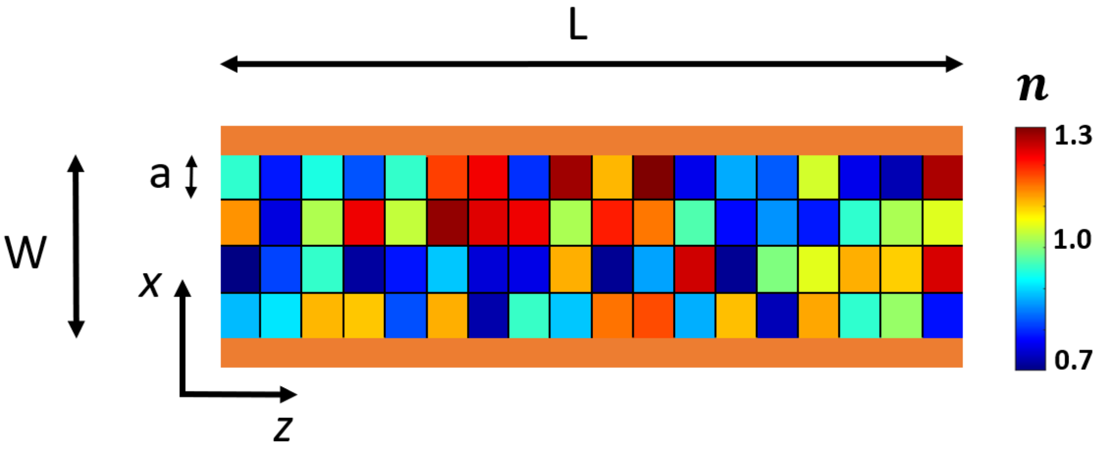

Model sample used in numerical simulations with length L, width W, and cell size a. The sample is bounded with perfect reflectors (orange) along the transverse boundaries in the z-direction, is connected to semi-infinite leads at the input and output surfaces along the x-direction and is constant in the y-direction.

Fig. S8 | Noise in transmission eigenvalues in samples with different N.

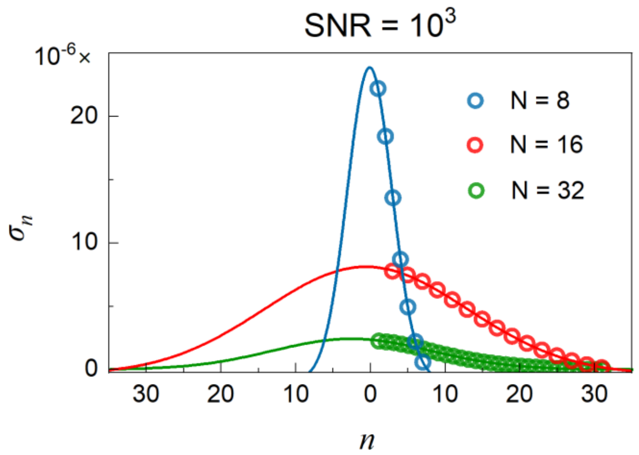

Simulations of samples in a narrow frequency range before the crossovers from $N = 7 \rightarrow 8$ (blue), $N = 15 \rightarrow 16$ (red), and $N = 31 \rightarrow 32$ (green). The Gaussian fit to σ_n is broader for larger N. The noise levels in both the highest and lowest TEs are lower for larger N.

Fig. S9 | Effect of spatial resolution on crossovers.

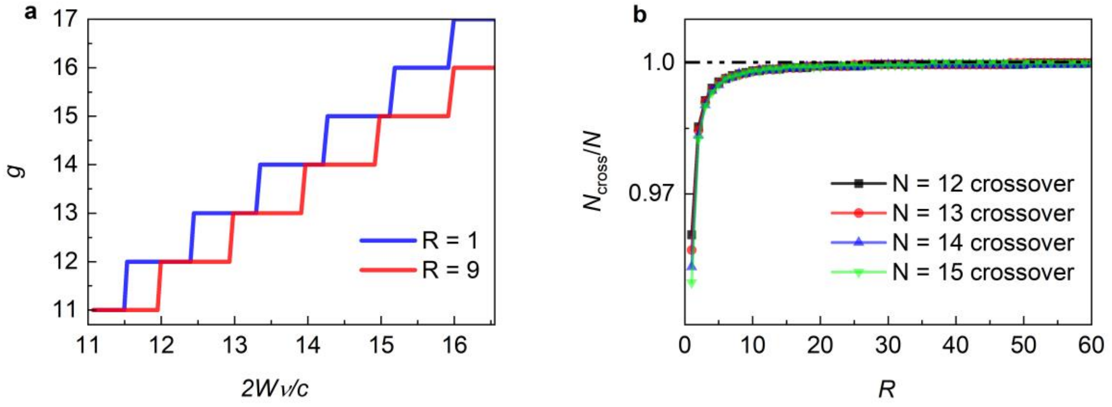

a, N_{cross} is the value of $2W\nu/c$ at a crossover as the width is increase in steps of a in an empty waveguide of lengths 100a and width 52a at two different spatial resolutions, $R = \lambda/2\pi a$. The crossovers approach integer values of N as R increases. **b,** Ratios of N_{cross} to integer values of N for a crossover from N to $N + 1$ versus R. Simulations in this paper were carried out for $R = 1$.

Fig. S10 | Measured conductance and density of states.

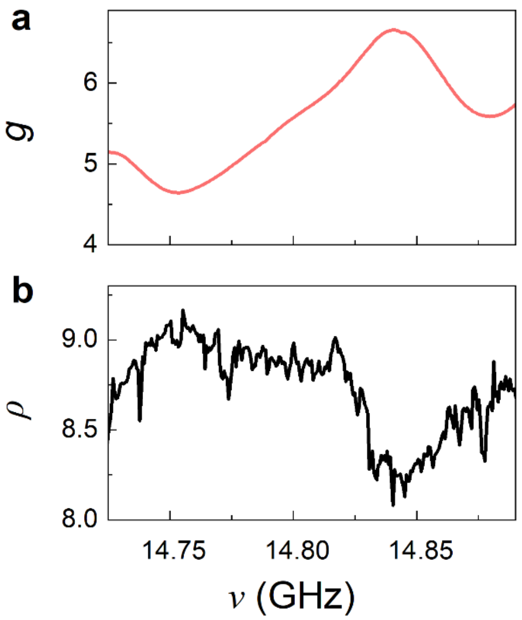

a, The measured conductance for $L = 23$ cm, as given in Fig. 1c, and **b,** the corresponding DOS, are compared. There is greater noise in the determination of the DOS, which is obtained from the transmission time, $\rho = \tau_T/\pi$, than of g, because the difference between the phase at two frequencies needs to be determined.

Fig. S11 | Correlation between transmission eigenvalues.

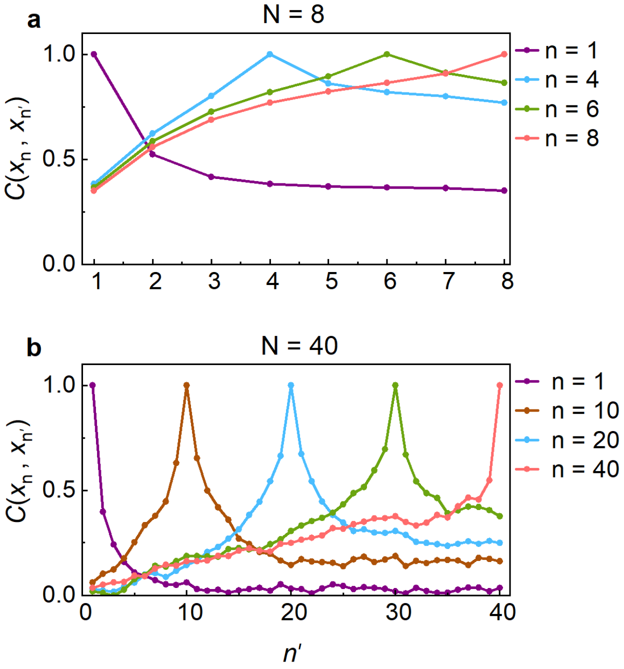

Correlations between various TEs for $N = 8$ (**a**) and $N = 40$ (**b**). The correlation between τ_N and the open channels is still appreciable for $N = 8$, but is significantly attenuated for $N = 40$.

Fig. S12 | Impact of TZs on conductance in single configurations.

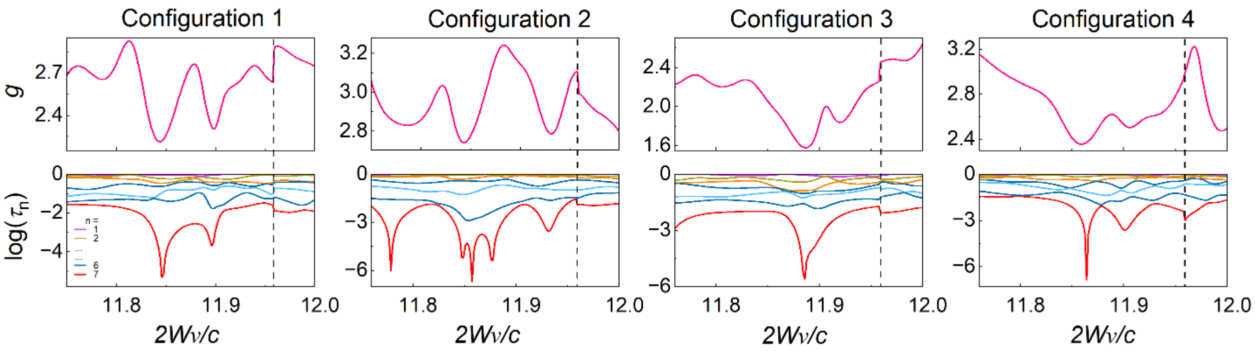

Simulations of spectra of the conductance (top panel) and transmission eigenvalues (bottom panel) for four different configurations with $L = 200a, W = 30a$ and disorder $\Delta n = 0.3$. For isolated TZs, the conductance dips at frequencies close to those at which TZs appear.

References

1. Pozar, D. M. *Microwave Engineering*. (John wiley & sons, 2011).
2. G. Metalidis. Electronic transport in mesoscopic systems. (2007).
3. Genack, A. Z., Huang, Y., Maor, A. & Shi, Z. Velocities of transmission eigenchannels and diffusion. *Nat. Commun.* **15**, 2606 (2024).
4. Huang, Y., Kang, Y. & Genack, A. Z. Wave excitation and dynamics in non-Hermitian disordered systems. *Phys. Rev. Res.* **4**, 013102 (2022).
5. Wang, J. & Genack, A. Z. Transport through modes in random media. *Nature* **471**, 345–348 (2011).
6. Kang, Y. & Genack, A. Z. Transmission zeros with topological symmetry in complex systems. *Phys. Rev. B* **103**, L100201 (2021).
7. Chabanov, A. A., Stoytchev, M. & Genack, A. Z. Statistical signatures of photon localization. *Nature* **404**, 850–853 (2000).
